# Innovative hematological strategies to combat maternal and child mortality in Africa: focus on anemia and sickle cell disease

**DOI:** 10.1097/MS9.0000000000004325

**Published:** 2025-11-18

**Authors:** Emmanuel Ifeanyi Obeagu

**Affiliations:** Department of Biomedical and Laboratory Science, Africa University, Mutare, Zimbabwe

**Keywords:** blood transfusion, child mortality, maternal mortality, point-of-care diagnostics, screening

## Abstract

Maternal and child mortality remain pressing public health challenges in Africa, driven by preventable hematological disorders such as anemia, hemorrhage, malaria-induced hemolysis, and hematologic complications of infections. These conditions are further compounded by limited diagnostic infrastructure and delayed access to advanced interventions. This narrative review explores emerging and innovative hematological strategies aimed at reducing maternal and child deaths across the continent. It highlights the role of novel diagnostic tools such as point-of-care hematology analyzers, genetic screening for inherited blood disorders, and rapid hemoglobin quantification in improving early detection and treatment. Advancements in iron supplementation, transfusion safety, and regenerative hematology, including the use of stem cell-based therapies, are discussed as transformative solutions. Moreover, the integration of digital health platforms and community-based blood surveillance systems offers new opportunities to strengthen maternal and neonatal care. By synthesizing current evidence, this review underscores the potential of hematology-centered innovations to close equity gaps, enhance survival outcomes, and accelerate progress toward the Sustainable Development Goals (SDG 3.1 and 3.2) in Africa.

## Introduction

Maternal and child mortality continue to represent some of the most critical public health concerns in sub-Saharan Africa, despite decades of global and regional interventions^[1,2]^. According to the World Health Organization (WHO), approximately 70% of global maternal deaths and over half of child deaths occur in Africa, primarily due to preventable or treatable causes^[3,4]^. Hematological disorders – including severe anemia, postpartum hemorrhage (PPH), malaria-related hemolysis, sickle cell disease (SCD), and nutritional deficiencies – are among the leading contributors to these deaths. These conditions not only compromise oxygen transport and hemostatic balance but also heighten susceptibility to infections and poor pregnancy outcomes^[5,6]^. Recent reviews and field studies emphasize that traditional interventions, though beneficial, have not sufficiently addressed the multifactorial nature of hematologic disorders in maternal and child health^[7-9]^. Factors such as delayed diagnosis, lack of blood transfusion services, inadequate prenatal screening, and limited access to hematological expertise further exacerbate mortality risks. The COVID-19 pandemic also disrupted essential health services, widening gaps in blood donation, laboratory support, and perinatal care^[10–12]^. Hence, there is a pressing need to adopt innovative, hematology-driven strategies that are sustainable, cost-effective, and contextually appropriate for African health systems^[13]^.

**HIGHLIGHTS**

- Innovative hematological strategies can reduce maternal and child mortality in Africa.
- Point-of-care diagnostics enable early detection of anemia and coagulopathies.
- Genetic screening improves management of sickle cell disease.
- Advanced transfusion systems and iron therapies enhance survival outcomes.
- Digital health and community engagement strengthen hematology-focused care delivery.

Emerging technologies and practices are beginning to transform the hematologic landscape in maternal and child care^[14]^. Point-of-care (POC) diagnostics, artificial intelligence (AI)-assisted screening, community blood donation networks, and portable transfusion devices are revolutionizing early detection and intervention^[15,16]^. Additionally, the integration of genomic medicine for identifying inherited blood disorders, regenerative stem cell approaches, and advanced iron supplementation methods holds promise for improving survival outcomes^[17,18]^. Importantly, these innovations must be accompanied by capacity building, policy reform, and equitable access initiatives to ensure that progress benefits even the most resource-limited communities^[19]^. This narrative review aims to synthesize current evidence on innovative hematological strategies designed to reduce maternal and child mortality in Africa. It explores diagnostic, therapeutic, and technological advances that are reshaping hematologic care, discusses implementation challenges, and highlights opportunities for integration into public health policy. By examining these multidimensional approaches, the review underscores how hematology can serve as a cornerstone for achieving maternal and child survival targets across the continent.

## Aim

The aim of this narrative review is to explore and synthesize current evidence on innovative hematological strategies designed to reduce maternal and child mortality in Africa. Specifically, it seeks to highlight recent advancements in diagnostic technologies, therapeutic interventions, and health system innovations that address hematologic disorders contributing to adverse maternal and neonatal outcomes. The review also aims to identify gaps in implementation, discuss opportunities for policy integration, and propose context-specific approaches to strengthen hematology-driven healthcare delivery and improve survival outcomes across the continent.

## Methods

This narrative review was conducted to provide a comprehensive synthesis of current literature on innovative hematological strategies aimed at reducing maternal and child mortality in Africa. A structured literature search was performed across major electronic databases, including PubMed, Scopus, Web of Science, and Google Scholar, covering publications from January 2000 to September 2025. Search terms included combinations of keywords such as “maternal mortality,” “child mortality,” “hematology,” “innovation,” “Africa,” “anemia,” “blood transfusion,” “genomic screening,” and “point-of-care diagnostics.” Both peer-reviewed articles and relevant grey literature (e.g., WHO reports, national health policy documents, and conference proceedings) were reviewed to capture a broad perspective. Studies focusing on Africa or presenting transferable innovations applicable to low-resource settings were prioritized. Inclusion criteria encompassed studies that discussed diagnostic, therapeutic, or technological hematologic innovations relevant to maternal or child health. Exclusion criteria included articles unrelated to hematological interventions or those without clear implications for mortality reduction.

Data were extracted and synthesized narratively, emphasizing emerging trends, intervention outcomes, and implementation challenges. The review adopted a thematic approach, categorizing findings into key domains such as diagnostic innovations, therapeutic strategies, transfusion and regenerative medicine, and health system integration. Although no formal quality assessment tool was applied due to the heterogeneity of included studies, emphasis was placed on methodological rigor, reproducibility, and contextual relevance. This methodological approach ensured a balanced and evidence-informed synthesis that aligns with the review’s objective of highlighting actionable hematological innovations capable of transforming maternal and child health outcomes in Africa.

### The burden of hematological disorders in Africa

Hematological disorders remain a major yet often underappreciated contributor to maternal and child mortality across Africa. Despite global progress in reducing mortality rates, Africa continues to bear a disproportionate share of deaths associated with preventable and treatable blood-related conditions. The WHO estimates that anemia affects over 45% of pregnant women and 60% of children under 5 years of age in sub-Saharan Africa, primarily due to iron deficiency, malaria, helminthic infections, and nutritional inadequacies. Severe anemia in pregnancy is strongly linked to increased risks of maternal death, PPH, low birth weight, and perinatal mortality^[20,21]^. PPH remains one of the leading causes of maternal death, accounting for nearly one-third of all maternal fatalities in Africa. The persistence of PPH-related mortality reflects the limited availability of blood transfusion services, delayed diagnosis of coagulopathies, and inadequate access to skilled obstetric care. Additionally, conditions such as SCD – which affects an estimated 300 000 newborns annually in Africa – further compound the burden by increasing susceptibility to infections, stroke, and pregnancy-related complications. Without early diagnosis and proper hematologic management, most affected infants do not survive beyond childhood^[22,23]^.

Infectious diseases with hematologic implications, such as malaria and human immunodeficiency virus (HIV), continue to exacerbate this burden. Malaria-induced hemolysis remains a major cause of severe anemia in children and pregnant women, while HIV-related thrombocytopenia and bone marrow suppression contribute to heightened bleeding and infection risks. The double burden of infection and hematologic dysfunction is further intensified by poverty, malnutrition, and fragile health infrastructure^[24]^. Moreover, limited access to diagnostic facilities, a shortage of trained hematologists, and inadequate laboratory capacity hinder the timely detection and management of blood disorders. Many health centers rely on basic clinical assessments rather than confirmatory hematological testing, leading to underdiagnosis and mismanagement. Blood shortages, poor donor mobilization systems, and transfusion-related risks also persist as systemic challenges^[25]^.

Addressing these hematologic determinants of maternal and child mortality requires a paradigm shift from reactive to preventive and technology-driven care. Strengthening diagnostic capabilities, integrating hematology into primary maternal and child health services, and expanding access to safe transfusion and genetic screening programs are essential steps. Recognizing the central role of hematologic health in survival outcomes is therefore critical to achieving equitable progress toward Sustainable Development Goal (SDG) targets for maternal and child mortality reduction in Africa^[26]^.

### Hematological innovations in maternal health

Hematological innovations are increasingly transforming maternal health care across Africa, offering new pathways to prevent and manage life-threatening conditions such as anemia, hemorrhage, and coagulopathies. Historically, maternal hematologic care in many African regions has been limited to symptomatic management due to resource constraints, inadequate screening, and lack of access to advanced diagnostics. However, recent progress in technology, clinical research, and health system integration has led to a shift toward preventive, precision-based, and community-oriented hematologic strategies^[27,28]^. One of the most significant advances has been the development and deployment of POC hematology analyzers, which enable rapid hemoglobin and hematocrit testing at antenatal clinics and community health centers. These portable devices allow for early detection of anemia and prompt initiation of iron or folate supplementation, even in rural areas with minimal laboratory infrastructure. Combined with mobile health (mHealth) applications, these tools facilitate real-time monitoring, data collection, and treatment follow-up, thereby strengthening continuity of care during pregnancy^[29]^.

Genetic and molecular screening for inherited blood disorders, such as SCD and thalassemia, has also gained prominence as an essential component of maternal health innovation. Integration of such testing into antenatal programs enables early counseling, carrier detection, and informed reproductive choices, reducing the risk of hematologic complications in pregnancy and neonates. Pilot programs in countries such as Nigeria and Ghana have demonstrated the feasibility and acceptability of antenatal sickle cell screening using affordable rapid test kits^[30]^. In addressing maternal anemia and iron deficiency, novel iron formulations – such as liposomal iron and intravenous ferric carboxymaltose – have shown superior absorption, better tolerance, and quicker hematologic recovery compared to traditional oral supplements. These innovations are particularly relevant for pregnant women with severe anemia who require rapid optimization of hemoglobin levels before delivery. Additionally, micronutrient fortification programs incorporating iron, folate, and vitamin B12 in staple foods have proven effective in population-level anemia reduction^[31]^.

Transfusion medicine has also evolved through the adoption of safer, more efficient blood collection, storage, and distribution systems. The establishment of community-based blood donor networks, drone-assisted delivery systems (as pioneered in Rwanda and Ghana), and improved blood screening technologies have significantly enhanced the availability of safe blood products during obstetric emergencies. Furthermore, research into synthetic and hemoglobin-based oxygen carriers holds promise for bridging transfusion gaps in remote or conflict-affected areas where conventional blood products are scarce^[32]^. Emerging evidence also supports the use of regenerative and stem cell therapies in managing refractory cases of PPH and hematopoietic dysfunction, though such approaches remain in early stages of implementation in Africa. Complementary innovations in digital health – such as telehematology platforms that link rural clinicians to diagnostic laboratories and specialists – further expand access to expert-guided care^[33]^. These hematological innovations represent a transformative shift in maternal health management from reactive interventions to proactive prevention and personalized care. To ensure sustainability, however, these technologies must be supported by strong health systems, adequate funding, skilled workforce development, and policy frameworks that promote equitable access. Strengthening hematology-centered maternal care can thus play a pivotal role in reducing preventable maternal deaths and achieving the targets outlined in the Sustainable Development Goals (SDG 3.1) for Africa (Table 1)^[34]^.

**Table 1:** Innovative hematological strategies aimed at reducing maternal and child mortality in Africa

Strategy/innovation	Target condition	Description	Implementation notes
Point-of-care hemoglobin analyzers (e.g., HemoCue, TrueHb)	Anemia in pregnancy and children	Portable devices for rapid hemoglobin/hematocrit testing	Effective in rural clinics; requires minimal training
Noninvasive hemoglobin meters	Anemia	Optical devices for quick hemoglobin estimation without blood draw	Reduces needle-stick injuries and improves patient acceptance
Rapid genetic screening for SCD/thalassemia	Sickle cell disease, hemoglobinopathies	Simple, low-cost point-of-care kits for newborn and antenatal screening	Requires counseling and linkage to care pathways
Intravenous iron formulations (ferric carboxymaltose, iron sucrose)	Moderate-to-severe anemia in pregnancy	Rapid correction of iron deficiency when oral iron fails	Requires trained personnel and monitoring for infusion reactions
Micronutrient fortification programs	Iron and folate deficiency	Enrichment of staple foods with iron, folate, and vitamin B12	Sustainable, population-level approach; needs regulatory support
Improved blood transfusion systems	Postpartum hemorrhage, severe anemia	Community donor networks, safe storage, drone-assisted blood delivery	Requires logistics coordination and quality assurance
Synthetic/hemoglobin-based oxygen carriers	Severe anemia, hemorrhage	Alternative oxygen-carrying solutions when blood is unavailable	Still limited in Africa; regulatory approval needed
Digital health platforms and telehematology	Maternal and child care, anemia monitoring	mHealth apps, EHR integration, remote specialist consultation	Requires internet access and training of health workers
Regenerative and stem cell therapies	Refractory anemia, SCD complications	Autologous stem cell transplantation or gene-modified cell therapy	High cost, limited centers; mainly research-based in Africa
Community health worker engagement	Hematological disorder education and early detection	Training CHWs to screen, educate, and refer pregnant women/children	Effective in culturally sensitive messaging and rural coverage

### Hematological approaches for child health

Addressing child mortality in Africa requires a concerted focus on hematological health, as many pediatric deaths are linked to blood-related disorders such as anemia, malaria-induced hemolysis, SCD, and infections leading to sepsis and disseminated intravascular coagulation. Innovative and targeted hematological strategies are now playing a transformative role in improving child health outcomes across the continent^[35]^. A cornerstone of pediatric hematological care is the early diagnosis and treatment of anemia, particularly iron deficiency anemia, which remains the most prevalent hematological disorder among African children. Recent strategies have emphasized mass supplementation programs using micronutrient powders, fortified foods, and therapeutic iron formulations. Intravenous iron and erythropoietin are now selectively used in severe cases, especially in children with chronic illnesses or those undergoing frequent transfusions.

These interventions not only restore hemoglobin levels but also support cognitive development and immunity, essential for early childhood growth and survival^[36]^. Neonatal and pediatric screening for SCD is another major hematological advance. Many countries have begun integrating newborn screening programs into primary healthcare systems, allowing for early identification and enrollment into comprehensive care programs. Hydroxyurea therapy, which stimulates fetal hemoglobin production and reduces vaso-occlusive crises, has gained wider acceptance and proven effectiveness in reducing hospitalizations and improving survival rates. In addition, family education and genetic counseling are increasingly promoted to reduce the burden of inherited hemoglobinopathies through informed reproductive choices^[37]^.

Malaria, a leading cause of anemia and mortality in African children, has also been targeted through hematologically focused strategies. In areas of high transmission, intermittent preventive treatment in infants using antimalarial drugs, combined with prompt treatment of malaria-related anemia, has been shown to significantly reduce fatal outcomes. Blood transfusion protocols for children with severe malaria-associated anemia have been updated to ensure timely and appropriate support, particularly in rural settings where delays often prove fatal^[38]^. Innovative diagnostic technologies are further strengthening pediatric care. Devices that assess hemoglobin, reticulocyte counts, and coagulation parameters using minimal blood volumes are now available and suitable for pediatric populations.

These tools, often embedded in mobile clinics or outreach programs, support early detection and prompt management of hematological abnormalities. Moreover, dried blood spot technology is enabling expanded screening for conditions such as thalassemia and glucose-6-phosphate dehydrogenase deficiency, both of which can complicate pediatric care if undetected^[39]^. Efforts to expand access to safe blood for pediatric transfusions are another crucial aspect of improving child survival. Many African nations have prioritized pediatric emergency transfusion services, with some countries establishing dedicated blood banks for children. Innovations such as blood grouping microcards, satellite blood fridges in rural health facilities, and community-based donor recruitment have helped reduce transfusion delays and prevent avoidable deaths from hemorrhage and severe anemia^[40]^.

**Figure 1.**
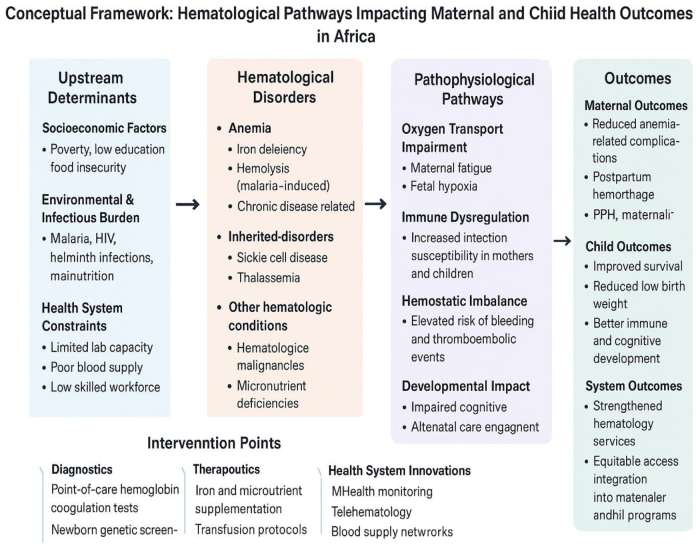
Conceptual framework: hematologic pathways impacting maternal and child health outcomes.

Hematological approaches also play a role in combatting sepsis and bleeding disorders in children. Platelet transfusion protocols, coagulation factor concentrates, and the use of antifibrinolytic agents like tranexamic acid are becoming more widely available and used in pediatric emergency and surgical settings. Where congenital bleeding disorders such as hemophilia exist, expanded access to prophylactic factor therapy has helped reduce joint damage, disability, and early death^[41]^. Capacity building through specialized training in pediatric hematology is improving the quality of care delivered. Initiatives that train healthcare providers in blood disorder recognition, transfusion safety, and the use of hematologic diagnostics have yielded positive results. Multidisciplinary teams – including pediatricians, hematologists, and lab scientists – are now more commonly involved in child health strategies, ensuring that hematologic care is both integrated and responsive to clinical needs (Fig. 1)^[41]^.

### The role of technology in hematological innovations

Technology is playing a transformative role in advancing hematological care, particularly in resource-constrained regions of Africa where maternal and child mortality remains a significant public health challenge. By bridging gaps in diagnostics, treatment, data management, and healthcare access, technological innovations are reshaping the landscape of hematological services and supporting timely, accurate, and life-saving interventions^[42]^. One of the most impactful technological breakthroughs in hematology is the development of POC diagnostic devices. These portable tools allow for rapid, on-site assessment of key hematological parameters such as hemoglobin levels, white blood cell counts, and coagulation status, significantly reducing diagnostic turnaround times. Devices like HemoCue and smartphone-linked hemoglobinometers have proven particularly useful in rural and under-resourced areas, where access to laboratory infrastructure is limited.

Their ease of use and minimal training requirements make them ideal for frontline healthcare workers managing pregnant women and children at risk of anemia or other blood disorders^[43]^. mHealth applications have also become central to improving patient monitoring and engagement. These digital platforms support remote tracking of patient data, medication adherence, appointment scheduling, and health education. For instance, women with high-risk pregnancies complicated by sickle cell anemia or other hematological conditions can now receive regular health reminders and communicate with healthcare providers via mobile apps. In pediatric care, digital monitoring of children with SCD facilitates early intervention during vaso-occlusive crises and ensures continuity of care, even in hard-to-reach communities^[44]^.

Technological advancements in telemedicine are further enhancing hematology service delivery. Through virtual consultations, specialists can support rural clinicians in managing complex hematological cases, provide real-time decision support, and guide emergency interventions. Telehematology services also enable centralized interpretation of blood smears, bone marrow aspirates, and other diagnostic tests, mitigating the shortage of trained hematopathologists in many African regions. This virtual extension of expertise helps to democratize access to specialized care and reduce preventable complications and deaths^[45]^. Moreover, AI and machine learning are being increasingly applied to hematology. These technologies support automated image analysis for blood cell morphology, predictive modeling of disease progression, and identification of patients at risk of adverse outcomes.

AI-powered algorithms can analyze large datasets from blood banks, hospitals, and screening programs to optimize resource allocation, detect emerging trends, and support national surveillance systems aimed at reducing maternal and child mortality^[46]^. Cloud-based electronic health record (EHR) systems represent another innovation enhancing hematologic care delivery. EHRs enable longitudinal tracking of patient data, facilitate referrals across different levels of care, and promote evidence-based decision making. Integration of hematological data into digital health platforms ensures that interventions such as blood transfusions, iron supplementation, or hydroxyurea therapy are documented and optimized. In maternal health, this allows clinicians to closely monitor women with chronic anemia or clotting disorders, reducing risks during delivery and postpartum^[47]^.

Blood safety technologies have also made significant progress, including nucleic acid testing for transfusion-transmissible infections, pathogen inactivation techniques, and barcoding systems for blood bag tracking. These innovations are vital for protecting both mothers and children who require frequent or emergency transfusions. With the advent of digital inventory systems, blood banks can better manage stock, reduce wastage, and prioritize urgent requests from maternity and pediatric wards^[48]^. Technological innovation is strengthening training and capacity-building in hematology through online learning platforms, virtual simulations, and e-mentorship programs. These tools allow health workers to stay updated on the latest clinical protocols, diagnostic methods, and transfusion safety practices. Virtual classrooms and mobile learning apps are increasingly used to equip healthcare providers in remote settings with the skills needed to manage complex hematological conditions confidently^[49]^.

### Strengthening healthcare systems for hematological care

Enhancing the capacity of healthcare systems to deliver effective hematological care is pivotal to addressing maternal and child mortality in Africa. A robust, integrated healthcare infrastructure ensures timely diagnosis, treatment, and follow-up of hematological conditions such as anemia, hemoglobinopathies, and coagulation disorders, which disproportionately affect vulnerable populations on the continent. Strengthening these systems involves multifaceted efforts that include workforce development, policy alignment, infrastructure investment, and efficient service delivery models^[50]^. A fundamental step in building strong hematological care systems is addressing the shortage of trained healthcare personnel. There is a critical need to expand the number of hematologists, transfusion medicine specialists, laboratory technologists, and nurse practitioners with competencies in blood disorders. Governments and academic institutions must collaborate to introduce hematology-specific training modules into medical and nursing curricula and to provide continuous professional development opportunities.

Furthermore, incentivizing rural deployment of skilled personnel through scholarships, housing, or career advancement pathways can help close the urban–rural divide in access to care^[51]^. Infrastructure enhancement is another cornerstone of a strengthened healthcare system. Many healthcare facilities across Africa lack adequately equipped laboratories, reliable power supply, or proper storage for blood products – all of which are essential for safe and effective hematological services. Investment in basic laboratory infrastructure for complete blood counts, hemoglobin electrophoresis, and coagulation studies, along with reliable supply chains for consumables, is urgently required. Additionally, ensuring the availability of blood products through well-managed and adequately resourced blood banks can significantly reduce mortality during obstetric hemorrhages and pediatric crises^[52]^.

Health policy and governance structures also play a critical role. Ministries of health must prioritize hematological diseases in national health strategies and align them with maternal and child health agendas. This includes setting national guidelines for anemia management in pregnancy, standardizing protocols for newborn screening of SCD, and incorporating hematological indicators into national health information systems. Strengthened governance fosters accountability, resource mobilization, and sustainable program implementation^[53]^. Efficient service delivery models are needed to ensure continuity and comprehensiveness of hematological care. Integrating hematological services into primary healthcare, maternal and child health clinics, and community health programs can improve early identification and management of blood disorders. Decentralization of services to community health centers, coupled with robust referral systems, ensures that patients can access specialized care when needed without excessive delays or financial burden^[54]^. Community engagement and health education are also essential. Cultural beliefs and limited health literacy often hinder early health-seeking behavior and adherence to treatment.

Community-based awareness campaigns can demystify blood disorders, promote early antenatal registration, and encourage uptake of interventions like iron supplementation and childhood screening. Training community health workers (CHWs) to identify and refer patients with suspected hematological conditions can enhance the reach and effectiveness of healthcare systems^[55]^. Data management and health information systems must be optimized to support evidence-based decision making. The systematic collection, analysis, and use of hematological data – including anemia prevalence, transfusion rates, and hemoglobinopathy screening outcomes – can inform policy decisions, resource allocation, and program evaluation. Investment in digital health infrastructure ensures real-time monitoring, enhances surveillance, and supports quality improvement initiatives^[56]^. Partnerships between governments, academic institutions, nongovernmental organizations, and international health agencies are vital to strengthening hematological care systems. Multisectoral collaborations can leverage funding, research, and technical expertise to build capacity, scale innovations, and sustain impact. These alliances are instrumental in establishing centers of excellence, conducting operational research, and advocating for equitable access to hematological care across the continent^[56,57]^.

### Collaborative efforts for maternal and child health

Collaboration between governments, nongovernmental organizations, and international health bodies is vital for reducing maternal and child mortality in Africa. By working together, these stakeholders can ensure that innovative hematological strategies are implemented effectively. Governments must prioritize hematological care in maternal and child health policies, ensuring that sufficient funding and resources are allocated to tackle anemia, SCD, and other blood disorders^[58,59]^. Additionally, partnerships with international organizations, such as the WHO, the United Nations, and the Global Fund, can provide the technical support, research, and funding necessary to scale up innovative hematological strategies. Through collaborative efforts, Africa can move toward achieving the SDG of reducing maternal and child mortality by 2030^[60–63]^.

## Conclusion

Reducing maternal and child mortality in Africa requires an integrated approach that recognizes hematological health as a central determinant of survival. This review highlights that innovative hematological strategies – ranging from POC diagnostics and genomic screening to improved transfusion systems and novel iron formulations – are redefining the prevention and management of life-threatening conditions such as anemia, hemorrhage, and inherited blood disorders. These innovations, when adapted to local contexts, have the potential to close long-standing health equity gaps and strengthen resilience within maternal and child health systems.

However, realizing their full impact demands more than technological advancement. Sustainable success depends on robust policy implementation, local capacity building, and investment in laboratory infrastructure and supply chains. Collaboration between governments, academic institutions, and international health partners is essential to ensure that hematologic innovations are affordable, accessible, and aligned with national health priorities. Moreover, integrating these interventions into routine antenatal and child health programs will ensure continuity of care and early intervention for at-risk populations. Prioritizing hematology-driven innovations offers a practical and evidence-based pathway to accelerate progress toward the SDG – particularly SDG 3.1 and 3.2, which target reductions in maternal and child mortality. By strengthening research, fostering equitable access, and promoting context-sensitive implementation, Africa can transition from managing preventable hematologic complications to achieving sustained maternal and child health gains.
